# Natural Aging of Ethylene-Propylene-Diene Rubber under Actual Operation Conditions of Electrical Submersible Pump Cables

**DOI:** 10.3390/ma14195520

**Published:** 2021-09-24

**Authors:** Freddy Ignacio Rojas Rodríguez, José Roberto d’Almeida Moraes, Bojan A. Marinkovic

**Affiliations:** Department of Chemical and Materials Engineering, Pontifical Catholic University of Rio de Janeiro (PUC-Rio), Rio de Janeiro 22453-900, Brazil; freddyrojas@aluno.puc-rio.br (F.I.R.R.); dalmeida@puc-rio.br (J.R.M.d.)

**Keywords:** ethylene-propylene-diene monomer, activation energy of degradation, hardness, Fourier-transform infrared spectroscopy, crosslink fraction, absorption

## Abstract

Ethylene-propylene-diene monomer (EPDM) rubbers used in electric submersible pump (ESP) cables were analyzed after being aged in actual operation conditions in oil wellbores. These rubbers constitute the insulation and jacket layers of the ESP cables. EPDM rubbers from four different cables operating during different time intervals (2 and 4.8 years) at different depths (from 760 to 2170 m) below sea level were studied. To verify the effects of the long exposure on the rubber performance, thermal analysis was performed to determine the thermal stability and activation energy of degradation. In addition, structural analysis, through vibrational spectroscopy and crosslinking fraction assessment, was carried out. The mechanical properties of the aged rubbers were inferred through the measurement of hardness, while the absorption of a service fluid was studied by gravimetry. The results showed only minor changes in the thermal, structural, mechanical and barrier properties of the EPDM-based ESP cable layers. It is suggested that the thermo-oxidation mechanism followed by chain scission does not have a role in the degradation of EPDM within the aged ESP cables, and no sign of variation of crosslink fractions has been encountered. Therefore, it was concluded that EPDM-based layers seem not to be weak links in the configuration of modern ESP systems.

## 1. Introduction

Electrical Submersible Pumps (ESP) have been used in the petroleum industry for artificial oil lifting from wellbores, while ESP cables serve to supply electrical power to the pumps [1]. ESP cable configuration is complex and composed of four different layers (insulation layer, covering, jacket and metal armor) surrounding three cooper wires carrying electrical current. Two of the layers, insulation and jacket, are frequently made from EPDM rubber with mutually distinct functions and properties. The insulation layer is dedicated to envelop copper wires and, therefore, has to present a high dielectric strength [2], in addition to resistance to crude oil and gas penetration. The jacket, on the other hand, provides mechanical protection to inner layers and has to be resistant to percolation of fluids.

EPDM rubber is widely used in industrial applications due to its low electrical and thermal conductivities, high weather, ozone and polar substances resistance and low adsorption of fluids. It is used in the automotive industry as window seal [3,4], in water supplying systems as water seal rings [5], in nuclear power plants within electrical cables as insulating material [6,7] and in the oil and gas industry [8].

There are various studies on EPDM degradation, generally performed through artificial aging [5,7,9,10,11]. However, it is also essential to evaluate degradation of EPDM during aging under operating conditions in different industrial applications.

It is also relevant to highlight that EPDM can be used with or without fillers, depending on its specific use, although the content of EPDM-based parts is commonly a trade secret in industrial applications. Among the fillers, carbon black is frequently used in EPDM formulations, since it improves tensile strength and conductivity, although decreases elongation [12] while, at the same time, increases the resistance to UV radiation [13]. Incorporation of clays additionally increases the dielectric constant of EPDM [14].

Seo et al. [6] performed a thorough study on artificial aging of EPDM, containing more than 30 wt% of fillers, used as an insulator in electrical cables in nuclear power plants. The authors evaluated their aging degradation and lifetime after accelerated aging at temperatures between 140 and 170 °C and, also, after a loss of coolant accident (LOCA), when temperatures and pressures can reach values higher than 180 °C and 4 atm. No radiation was used for aging purposes.

The authors found [6] that temperatures higher than 140 °C are particularly harmful and can reduce the lifetime almost 10 times, as determined during aging at 170 °C. In addition, activation energies of degradation of EPDM showed reduction between 15 and 25% after LOCA conditions, in comparison to EPDM before LOCA, depending on the experimental data used for their calculation. In accordance with the authors, the reduction of activation energy was an indication of polymer degradation through mechanisms such as thermo-oxidation and chain scission. Interestingly, Fourier-Transform InfraRed spectroscopy (FTIR) did not reveal any change in EPDM spectra, before and after LOCA conditions, despite the fact that C=O and C=C bands should be expected in accordance with the degradation mechanisms and despite the significant decrease in mechanical properties.

Zhao et al. [15] studied EPDM degradation under artificial accelerated weathering conditions, using high humidity (65%) and temperature (55 °C), rain and light exposure intervals and concluded that in such severe conditions, degradation of EPDM is rapid along the first 12 days.

Šarac et al. [7] performed artificial aging of EPDM using different temperatures and radiation doses. Although this study is not directly connected to the operation conditions of EPDM in ESP cables, it is still a rare study of artificial EPDM aging. They reported that at room temperature, ultimate tensile stress increased firstly with an increase in absorbed radiation doses until 600 kGy and decreased for the higher doses. Such a behavior of the ultimate tensile stress has been explained as the interplay between crosslinking, predominant at lower doses, with the chain scission mechanism, dominating at higher radiation doses.

Wang et al. [9] reported that the tensile strength and elongation at break decreased with time of artificial aging at seawater conditions, while hardness increased. The authors pointed to chain scission as the mechanism causing degradation of EPDM under seawater conditions.

Nakamura et al. [5] reported a rare study of natural aging of EPDM under actual operation conditions. They investigated degradation of EPDM used in seal rings in drinking water supplying systems, since degradation fragments might contaminate water. These EPDM seals have been naturally aged for 3 years in contact with the water supplied in Osaka city, Japan, at temperatures varying between 20 and 45 °C. The authors encountered a decrease in hardness of about 10% and almost 40% reduction in crosslinking density in comparison to unused EPDM seals. Scanning Electron Microscopy (SEM) evidenced a significant degradation of naturally aged EPDM seals through observation, since degradation fragments might contaminate water. Degradation mechanisms were attributed to thermo-oxidation of EPDM and chain scission. FTIR spectrum of aged seals exhibited vibrational bands at 1640 and 1720 cm^−1^ identified as C=C and C=O bonds, respectively, while in the FTIR spectrum of unused seals, these bands were absent. It is worth noting that chain scission is responsible for the formation of C=C bonds inside EPDM.

Li et al. [16] studied thermo-oxidative and compressive stress-thermo-oxidative aging of EPDM and concluded that activation energy decreases with the increase in compressive stress. This indicated that compressive stress contributes to accelerate degradation mechanisms in EPDM.

Awwad et al. [11] evaluated the aging of EPDM exposed to a NaOH solution (25 wt% NaOH) at 38, 54 and 77 °C, as well as to water at 77 °C for 12 months. Tensile strength decreased as a function of temperature and exposure time. The longer the time and the higher the temperature, the greater the decrease in mechanical properties. This behavior was attributed to the NaOH solution penetration inside EPDM samples.

ESP cables are currently exchanged in regular time spans to avoid cable deterioration and, consequent, non-planned interruption of oil production. Despite their importance in the petroleum industry, there is a complete lack of knowledge on natural or artificial aging of EPDM components, used for fabrication of insulation and jacket layers, inside ESP cables, as far as the authors are aware of. In addition, ESP recently started to employ permanent magnet motors, increasing significantly their service life time [1]. Therefore, the question has been raised if ESP cables become weak links inside the ESP system.

In order to elucidate changes of physico-chemical properties of EPDM used for fabrication of insulation and jacket layers, ESP cables naturally aged inside oil wellbores in actual operation conditions over 738 (~2 years) and 1752 days (~4.8 years) at different depths, with respect to sea level, were studied and compared with a virgin ESP cable.

The principal goal of this research was to evaluate the extent of degradation of insulation and jacket EPDM-based layers during actual operation conditions of ESP cables by studying their hardness, thermal stability, activation energy of degradation, chemical structure (through vibrational spectroscopy and crosslink fraction) and barrier capacity. The filler composition was also elucidated for both layers.

This work presents, as a novelty, the natural aging study of EPDM layers (insulation and jacket) within ESP cables after their use under actual service conditions and intends to evaluate whether these cables can become the weak link in new ESP systems, in which pumps with much greater durability are being used. This knowledge can be used as a guide for avoiding unscheduled interruptions of oil production and, therefore, for minimization of operating costs.

## 2. Experimental

### 2.1. Materials

Five ESP cables were kindly supplied by Equinor Brasil Energia Ltd. (Rio de Janeiro, Brazil). Two ESP cables (denoted, A and B) spent 738 days (~2 years) in real operation conditions at the depths of 917 and 1927 m, respectively. Two other ESP cables (denoted, C and D) spent 1752 days (~4.8 years) in real operation conditions at the depths of 760 and 2170 m, respectively. The temperatures of ocean water at the depths between 760 and 2170 m are approximately between 8 and 4 °C, while the pH of liquid in contact with ESP cables was ~8.

It is relevant to notice that the ESP cables A and B operated in a different wellbore from that of the cables C and D. The fifth cable, denoted E, was a virgin ESP cable. The metallic armor was made from galvanized steel for all studied ESP cables. A schematic draw of ESP cable configuration, with its principal layers, is presented in the Supporting Information, Figure S1.

In accordance with the datasheet of the two EPDM-based layers, insulation and jacket, these were made from EPDM DL90 and EPDM CL-185, respectively.

### 2.2. Characterization Techniques

Fourier Transform InfraRed spectroscopy (FTIR) was performed on a PerkinElmer Spectrum Two FT-IR-ATR spectrometer. Small quantities of insulation and jacket layers, from five ESP cables, were used for the analyses without any additional treatment. Spectra were recorded in the range from 400 to 4000 cm^−1^, with a resolution of 4 cm^−1^ and 20 scans.

Thermogravimetric analyses (TGA) were performed using a PerkinElmer Simultaneous Thermal Analyzer, model STA 6000 (PelkinElmer, Beaconsfield, England). Two different experimental conditions were used to analyze EPDM samples, regardless of the layer.

Under **condition I**, 10–15 mg of EPDM were heated under N_2_ flow (20 mL min^−1^), at heating rates of 5, 10, 15, 20, 25, 30 and 40 °C min^−1^ in the temperature range of 30–570 °C. Therefore, 7 runs were carried out per EPDM from each cable and from each EPDM layer (insulation and jacket). This totalized 70 runs carried out under **condition I**.

In the experiments using **condition II**, 10–15 mg of EPDM were heated from 30 to 570 °C under N_2_ flow (20 mL min^−1^), at a heating rate of 25 °C min^−1^, while from 570 to 930 °C gas flow was changed to synthetic air (20 mL min^−1^), while the heating rate was kept the same. This totalized 10 experiments, considering both EPDM layers, performed under **condition II**.

Crosslink fractions of EPDM from insulation and jacket layers were calculated following the experimental and data analyses methods described in ASTM D 2765-01 [17,18]. It is relevant to mention that this ASTM standard considers EPDM exclusively part of the total EPDM formulation (consisting of EPDM matrix and organic and inorganic fillers and additives) for calculation of crosslink fractions. At least three measurements were performed per EPDM sample.

The Shore D hardness test was performed with a Digimess durometer, based on the ASTM D 2240-15 standard.

For the insulation layer, cylindrical specimens were prepared and measurements were taken along the length of the specimen, as well as on the cross section, along the perimeter. For the measurement of hardness in the cross section, the specimens were only 50 mm long, in order to avoid any buckling effect during the measurement. On the other hand, the measurements along the length were made on 110 mm long specimens. The copper wires were not removed from the cylindrical specimens to avoid damaging the insulation EPDM layer. Nine measurements were taken along the length of the samples and eight measurements along the perimeter. Three samples were used per cable. Therefore, each data point (hardness of each EPDM specimen) consists of an average of 27 measurements for the hardness measured along the length of each cable, and 24 measurements for the hardness measured along the perimeter of each cable.

For the jacket layer, tests were performed on the cross section of cylindrical specimens cut from ESP cables. Four specimens were machined from each cable and measurements were carried out on both sides of the samples (top and bottom faces). On each face, 15 measurements were made, totalizing 120 measurements per cable.

Absorption tests were performed on the insulation and jacket rubber layers. The tests were based on ASTM D 570-98 standard, with the aim to determine the amount of fluid absorbed by gravimetry. For both layers, three samples from each cable—A to E—were immersed in the service Safe-Scan + Packer fluid (2.22% *v*/*v*) for up to 3777 h (~158 days). This is a protection fluid for ESP cables and was prepared with the formulation commonly used when the cables are in regular operation conditions. The samples of each cable were immersed in different containers to avoid any type of contamination/interference from samples coming from different cables. The samples were removed periodically from the containers and weighted within ±0.001 g, to determine the mass gain with respect to the immersion time.

The samples of the insulation layer, consisted of cylinders 60 mm long, from which the copper wires, that are surrounded by the insulation layer, have not been removed. This procedure was adopted considering that the removal of the copper wire produced too much damage in the rubber layer. Furthermore, any absorption of the fluid by the copper wire is not expected. Thus, the presence of the wire does not affect the measurement procedure. For the jacket layer, cylindrical specimens were used as well but 50 mm long. These specimens consisted of rubber segments, from which the external metallic armor and the insulation layer/copper wires assembly was removed.

## 3. Results

FTIR spectroscopy permits the identification of the thermo-oxidative degradation mechanism in EPDM during aging and also allows the identification of additives incorporated in EPDM formulations within the insulation and jacket layers.

**Figure 1:**
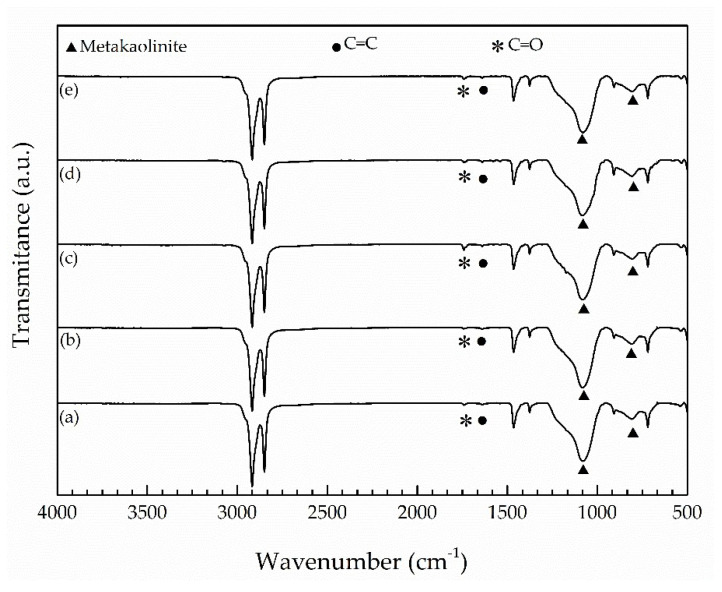
FTIR spectra of the insulation layer of cables (**a**) A; (**b**) B; (**c**) C; (**d**) D and (**e**) E. ▲ stands for metakaolinite, ● stands for C=C and * stands for C=O bands.

Figure 1a–e shows FTIR spectra of the insulation layer from the ESP cables A to E. The vibrational bands attributed to EPDM [6,19,20] are listed in Table 1. In addition to EPDM vibrational bands, all FTIR spectra exhibit the same group of additional bands attributed to metakaolinite [21], a product of thermal decomposition of kaolinite clay mineral at temperatures > 530 °C. It is worth noting also that the weak bands situated at 1720 and 1640 cm^−1^, attributed to carbonyl (C=O) and carbon–carbon double bonds (C=C) [5], respectively, were identified in the spectrum of the virgin ESP cable (Figure 1e), and also in the spectra of the naturally aged ESP cables during 738 and 1752 days at different depths (Figure 1a–d). The intensities of these two vibrational bands, which are the fingerprints of oxidation and chain scission degradation mechanisms of EPDM [5,6,22], apparently keep similar values in the virgin and naturally aged cables.

**Figure 2:**
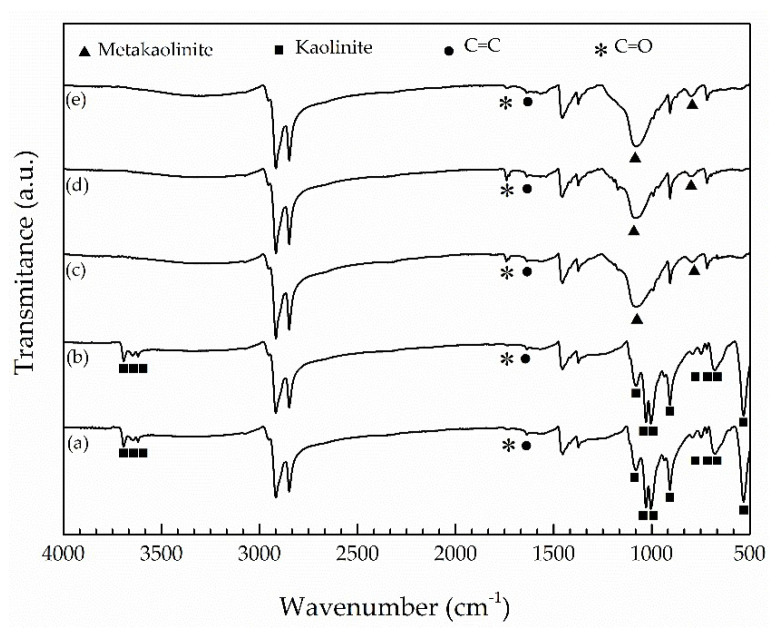
FTIR spectra of jacket layer of cables (**a**) A; (**b**) B; (**c**) C; (**d**) D and (**e**) E. ■ stands for kaolinite, ● stands for C=C and * stands for C=O bands, ▲ stands for metakaolinite.

FTIR spectra of EPDM composing a jacket layer of the ESP cables A to E are presented in Figure 2a–e. All spectra showed vibrational bands belonging to EPDM [6,19,20], as listed in Table 1. Furthermore, all spectra present additional bands similarly to that previously noted for the insulation layer. A peculiarity is that the EPDM from the cables A and B (aged for 738 days at the depths of 917 and 1927 m, respectively) presented vibrational bands of kaolinite [21], while EPDM from the cables C, D (aged for 1752 days at the depths of 760 and 2170 m, respectively) and E (virgin cable) presented vibrational bands of metakaolite [21]. As in the case of EPDM from the insulation layer, FTIR spectra of EPDM from the jacket also presented vibrational bands with similar intensities originated from carbonyl and carbon–carbon double bounds, regardless of whether the cable was virgin or naturally aged for 738 and 1752 days.

TGA has been conducted to identify degradation stages, to quantify content of additives, organic and inorganic, presented in EPDM formulation and, mainly, to calculate activation energy of degradation of EPDM, in order to shed more light on EPDM degradation, which might occur during natural aging.

**Figure 3:**
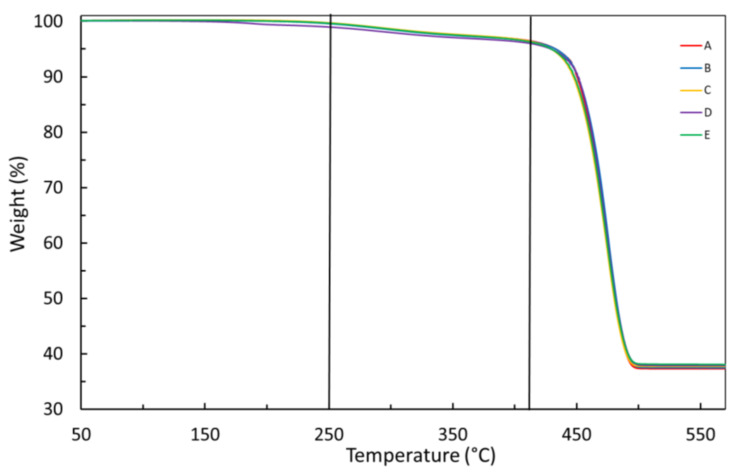
TGA curves of the insulation layer for virgin and naturally aged cables, under condition I (heating rate of 10 °C min^−1^). A–E stand for different ESP cables as denoted in the Section 2.

Figure 3 shows TGA curves of the insulation layer of the virgin ESP cable and for naturally aged cables under condition I. The weight loss stages and contents are very similar for all cables, virgin or aged (Table 2). Three stages of weight loss could be determined, at most, although in the first two, up to 410 °C, the weight loss is only slightly higher than 3 wt%. The small loss at the first stage until 250 °C (<1 wt%) may be attributed to humidity and some small organic molecules. The second stage loss, from 250 to 410 °C, is commonly ascribed to evaporation of extender oils, generally used in fabrication of EPDM to reduce viscosity and as fillers. In the third stage, from 410 to 520 °C, a weight loss, on average, as high as 59 wt% was recorded, a consequence of decomposition and volatilization of EPDM [6]. On average, about 37.5 wt% of the insulation layer remained after heating to 570 °C.

**Figure 4:**
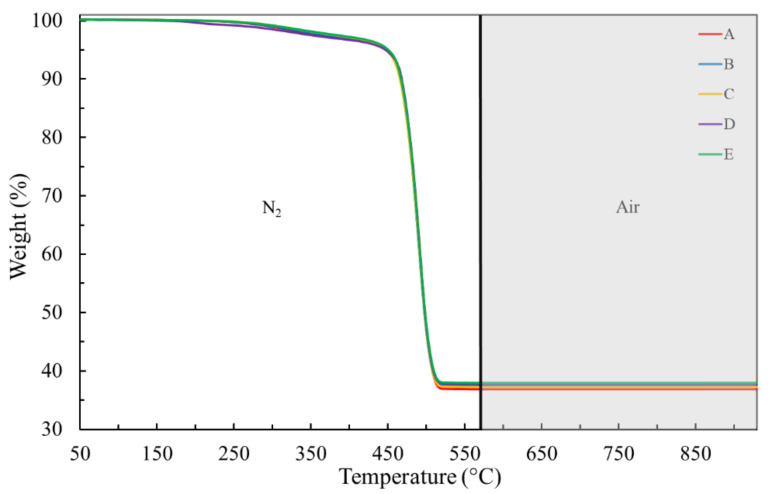
TGA curves of the insulation layer for virgin and naturally aged cables, under condition II (heating rate of 25 °C min^−1^). A–E stand for different ESP cables as denoted in the Section 2.

Under condition II, carried out until 930 °C, insulation layers of all five ESP cables showed the same mass loss stages and loss contents (Figure 4) as previously observed under condition I, indicating that the remaining mass of about 37.5 wt% is due to an inorganic filler such as metakaolinite, previously identified by FTIR (Figure 1).

**Figure 5:**
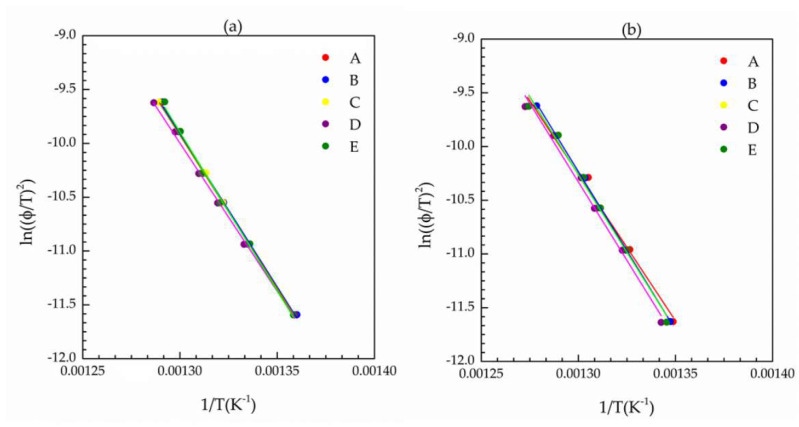
Kissinger plots (**a**) insulation layer (**b**) jacket layer. A–E stand for different ESP cables as denoted in the Section 2.

Interestingly, activation energies of degradation (E_a_) of insulation layers, calculated by the Kissinger model [23,24] for virgin and naturally aged cables are distributed within a few percentages around the value of the virgin cable of ~233 kJ mol^−1^ (Figure 5 and Table 3).

**Figure 6:**
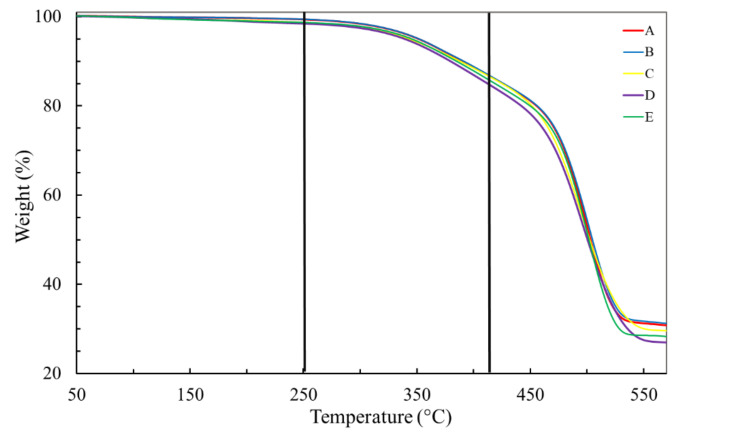
TGA curves of jacket layers for virgin and naturally aged cables, under condition I (heating rate of 25 °C min^−1^). A–E stand for different ESP cables as denoted in the Experimental section.

EPDM from the jacket layer also demonstrated three stages of weight loss under the **condition I** until 520 °C, while from 520 to 570 °C, no weight loss was detected (Figure 6). In the first stage (from 30 to 250 °C) the weight loss is low (Table 4), on average slightly higher than 1 wt%, and was attributed to water loss and, possibly, to the loss of some other low-weight organic molecule. During the second stage, from 250 to 410 °C, a weight loss of on average 14 wt% (Table 4) was detected and attributed to the loss of extender oils. In the last stage, from 410 to 520 °C, on average 56 wt% were lost (Table 4), which is in accordance with the well-known temperature range of EPDM decomposition [6]. Therefore, on average, 29 wt% of an inorganic filler remained present, and at least a portion of it can be ascribed to metakaolinite or kaolinite, depending on the cable, as pointed out by FTIR analyses (Figure 2).

**Figure 7:**
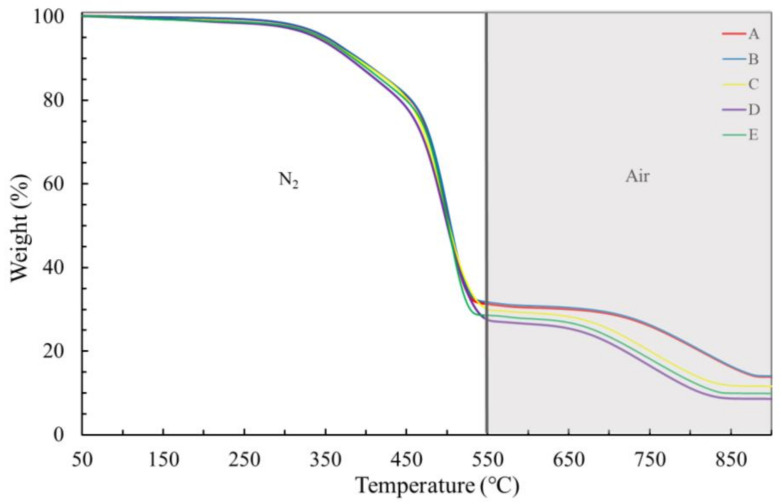
TGA curves of the jacket layer for virgin and naturally aged cables, under condition II (heating rate of 25 °C min^−1^). A–E stand for different ESP cables as denoted in the Experimental section.

In order to have a better understanding of filler types inside EPDM, composing about 29 wt% of the total EPDM formulation in the jacket layer, the **condition II** was applied. Figure 7 demonstrates that the switch of atmosphere at 570 °C, from inert (N_2_) to oxidative (synthetic air), is capable of identifying the presence of carbon black through the weight loss, between 700 and 800 °C, probably emitted as CO_2_. The weight lost due to carbon black is responsible for, on average, 18 wt%, while 8.5 to 14 wt% of residual material, depending on particular ESP cable, still presented at 930 °C, were due to metakaolinite and kaolinite fillers (Table 5).

Kissinger plots for EPDM from the jacket layer for all cables are shown in Figure 5 and the as-calculated values for the energy of activation are summarized in Table 3. It should be highlighted that the activation energies were calculated from seven experimental points (as it was the case for the insulating layer), offering a robust amount of data for their calculation.

The value for the energy of activation for degradation of EPDM, in jacket layer, for the virgin cable (E) is 237.83 kJ mol^−1^ and is higher than those calculated for the naturally aged cables (Table 3). EPDM naturally aged inside the cables B, C and D showed reduction of activation energy for their degradation of about 10% in comparison to the activation energy of virgin cable, while this reduction was about 5% for the EPDM from the cable A, which has been exposed to milder conditions in terms of time (738 days) and depth (917 m), in comparison to the other aged cables (B, C and D).

**Figure 8:**
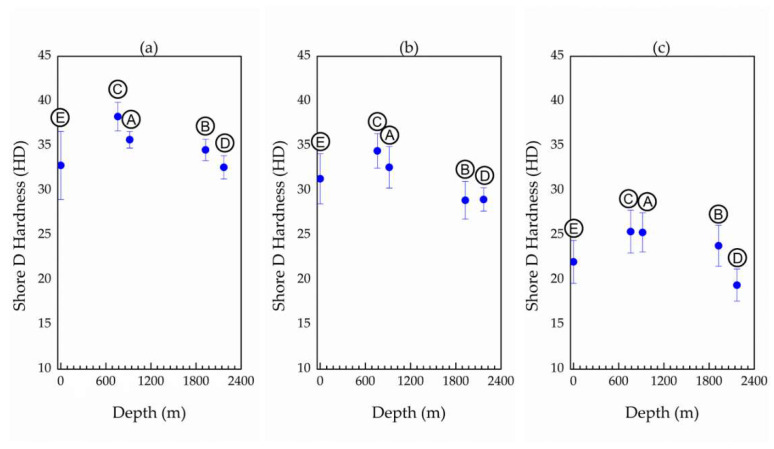
Hardness (**a**) along a longitudinal section of the insulation layer, (**b**) along a transversal section of the insulation layer, and (**c**) of the jacket layer. A–E stand for different ESP cables as denoted in the Experimental section.

The results of Shore D hardness tests on insulation layers of cables A to E show no significant variation in hardness as a function of service time or depth of operation, both in longitudinal and transversal modes (Figure 8a,b).

Comparing longitudinal vs. transversal hardness, there is a tendency for the values measured longitudinally to be slightly higher than those measured transversely. In addition to these small differences, it is reasonable to expect that both hardness values may deviate from the absolute values due to the shape/dimensions of the specimens.

The longitudinal measurements may present slightly overestimated values due to the small thickness of the rubber layer over the copper wire. Thus, it could be argued that the measured hardness value might be higher than expected due to the contribution of the copper substrate. On the other hand, the transverse hardness, also due to the small thickness of the rubber layer, may not be sufficiently rigid to confine the hardness dent and thus resulted in an underestimated hardness value.

If any of these experimental deviations are occurring, they are small and not significant for the general analysis, as can be seen from the results shown in Figure 8a,b.

The results of the Shore D hardness test of the EPDM jacket layer are shown in Figure 8c. Interestingly, their hardness was significantly lower (>20%) in comparison to the hardness of the insulating layer. However, similarly as found for the EPDM insulation layer, there was no significant variation in the hardness between the EPDM rubber of virgin and naturally aged cables.

Table 6 summarizes crosslink fractions of EPDM of the insulation layers, together with their standard deviations. The crosslink fractions of the virgin and naturally aged cables vary within the standard deviation. A maximum variation of 5.7% for the isolation layer and of 5.8% for the jacket layer were obtained.

The crosslink fractions of the jacket layers (Table 6) are significantly lower than the fractions calculated for the EPDM from the insulating layers. This is in accordance with their lower hardness, as previously measured by Shore D (Figure 8a–c). The crosslink fractions within the jacket vary in a narrow range close to 0.70, pointing out that this lower fraction interval is a characteristic of the EPDM used for fabrication of the jacket layer. No indication of the EPDM degradation in the jacket layer of naturally aged cables was evidenced by reduction, or increase, of crosslink content.

**Figure 9:**
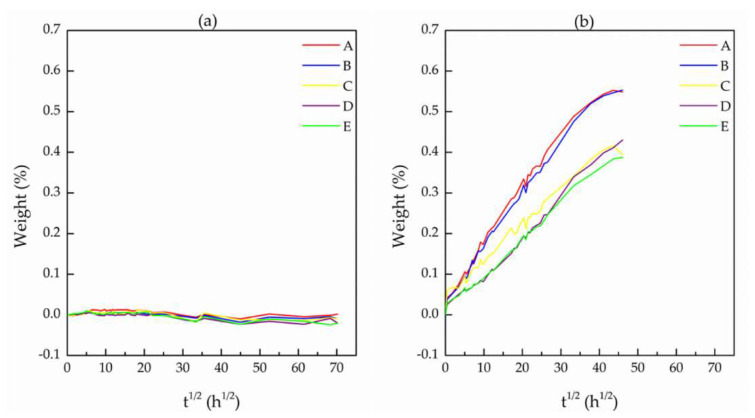
Adsorption plots of (**a**) insulation layer and (**b**) jacket layer. A–E stand for different ESP cables as denoted in the Experimental section.

Figure 9 shows the results of the absorption test for all analyzed samples. It can be observed that the insulation layer absorbed insignificant amounts of the fluid. The small variation found (±0.01%) was considered to be within the experimental error of the test, mainly due to the process of excess fluid removal from the sample surface at each weighing.

The results obtained for the jacket layer indicate a small absorption of up to about 0.55% after 158 days of immersing the samples in the Safe-Scan + Packer fluid. As can be seen, the absorption process did not reach complete saturation and there was a significant difference between the rubbers of cables A and B and C and D. Cables C and D present the same behavior found for the virgin cable (cable E). It should be remembered here that the cables A and B come from a different wellbore from that of the cables C and D. It is also important to note that the operating time of cables A and B (738 days) was much shorter than that of cables C and D (1752 days); however, there was greater absorption in cables A and B.

## 4. Discussion

Based on the intensities of the vibrational bands located at 1720 and 1640 cm^−1^, belonging to C=O and C=C vibrational bands, respectively, FTIR spectra of insulation and jacket layers (Figure 1 and Figure 2) did not support progress of thermo-oxidation and chain scission mechanisms of degradation during natural aging of ESP cables. Carbonyl groups and C=C bonds have already been introduced, in small amounts, during the fabrication process of EPDM formulations for both insulation and jacket layers, and FTIR was not capable of discerning further degradation of EPDM through these mechanisms during natural aging. Similarly, Seo et al. [6] did not find evidence from FTIR analyses on oxidation degradation after LOCA aging, through the appearance of the C=O band, although aged EPDM demonstrated significant reduction of mechanical properties, life time and even a decrease in activation energy of degradation at the level as high as 25%.

However, Nakamura et al. [5] were capable of observing the appearance of C=O and C=C vibrational bands in seal rings aged during 3 years in actual operation conditions, including temperatures as high as 45 °C. Li et al. [16] evidenced that the aging temperature is important for C=O formation on thermal-oxidation. They observed that the C=O band started to increase, in comparison to the unaged EPDM, only when aging temperature reached 70 °C. The same authors identified low intensity C=O vibrational bands also on unaged EPDM, corroborating the observation that some low extent of thermo-oxidation may already occur during fabrication. Wang et al. [25] also observed molecular chain degradation, bond breaking and EPDM oxidation, but they conducted their experiments at high temperatures (130, 145, and 160 °C).

Therefore, the absence of further oxidation in the EPDM inside insulation and jacket layers can be understood in terms of low temperatures of sea water (although not in direct contact with ESP cables), as reported in the Experimental section, and the lack of oxidation atmosphere at the aging depths, the conditions that do not promote thermo-oxidation.

In addition to the FTIR results, it is relevant that the activation energies of degradation for the aged insulation layer (Table 3), as calculated by TGA data through Kissinger analysis, are very close to the activation energy of the virgin cable (maximum variation of 3.9%), indicating the absence of degradation. However, activation energies of degradation for the jacket layer (Table 3) of aged cables showed a small decrease, suggesting that a degradation mechanism has been active, especially for the more severely aged cables B, C and D, which presented a reduction in activation energy as high as 10%. In comparison to the reduction of about 25% after LOCA aging reported by Seo et al. [6], the reductions of activation energy presented by EPDM from the jacket layer were moderate, situated between 10% (for more severe aging conditions in terms of time and depth) and 5% (for the least severe condition). The Ozawa–Flynn–Wall model, together with three other models, Friedman, combined kinetic analysis and correction of the combined kinetic analysis by isoconversion, were also used to estimate activation energy of EPDM degradation in the jacket layer. These results, to be present elsewhere, showed a similar trend of small or minor changes of activation energies with natural aging conditions.

The small decrease in activation energy of EPDM in the jacket layer, apparently, 5% to 10% as cited above, is not related to any kind of oxidation degradation as corroborated by FTIR but is probably connected to chemical degradation or hydrolysis induced by fluids and humidity, respectively, as inferred from the work of Woo et al. [26]. It is worth noting that the jacket layer is closer to the surface of the ESP cable and, therefore, is more susceptible to interaction with the chemical agents from the environment.

The higher hardness of EPDM in the insulation layer in comparison to the jacket (Figure 8) is due to higher, about 20%, crosslink fraction of EPDM inside the insulation layer (Table 6). Furthermore, EPDM formulation for the insulation layer is richer in inorganic content than for the jacket layer (Table 2, Table 4 and Table 5), which can additionally contribute to the higher hardness of EPDM in the insulation layer. The hardness of EPDM in the insulation layer is at the same level of Shore D scale reported by Kömmling et al. [27]. In addition, these authors found that the hardness of EPDM hardly changes with aging for temperatures ≤ 125 °C, while at the higher temperatures of aging (>125 °C), hardness increased several times, owing to additional crosslinking [28,29]. In accordance with the findings of Kömmling et al. [27], hardness of EPDM from insulation and jacket layers also hardly changed with natural aging, as observed from Figure 8.

In accordance with hardness measurements, crosslink fractions (Table 6) did not change significantly for the insulation or jacket layer. This would seem to show that EPDM in both layers did not suffer from a significant change in reticulation, as experimentally observed.

The higher absorption of jacket layer is attributed to fewer reticulated EPDM chains in this layer when compared to EPDM in the insulation layer (much more crosslinked). However, both EPDM layers absorbed an insignificant amount of Safe-Scan + Packer fluid, while it is worth noting that neither of the layers is actually in direct contact with environment liquids during service life time. Thus, both EPDM-based layers kept high barrier properties during aging.

The absorption tests (Figure 9) also seem to indicate an influence of the wellbore chemical environmental conditions on the absorption process. In fact, the interaction of EPDM rubber with organic compounds will vary depending on the type of compound, as expected but also with the volume fraction of different compounds in a mixture. For example, the mass uptake of aromatic hydrocarbons is higher than that of alkanes in EPDM rubber, and mixtures of these compounds increase the effect of absorption when the temperature is increased [30]. Since a wellbore contains a mixture of numerous hydrocarbons, deleterious synergistic effects can occur, which seems to be the case for the wellbore where cables A and B operated. Moreover, the deleterious effect of fluid absorption can be enhanced with the presence of CO_2_ in solution [31], and CO_2_ is one of the three most common gases found in oil wellbores, along with H_2_S and CH_4_ [32].

The present data strongly suggested that EPDM in insulation and jacket layers of ESP did not suffer, during prolongated aging, from thermo-oxidation degradation, which is detectable through the formation of the carbonyl group and chain scission. In addition, no sign of crosslink fraction change has been found for the studied EPDM layers even after being in operation for time periods as long as 4.8 years. It is worth noting that only EPDM from the jacket layer presented an indication of slight degradation through a low decrease in activation energy (10% at most). It can be speculated at this point that this reduction in activation energy is related to another type of aging, such as chemical degradation or hydrolysis, as pointed out by Woo et al. [26] in their study.

The most important findings of our study, although taken on a limited number of specimens, are that EPDM-based layers from ESP cable seem not to be weak links within the modern ESP systems with longer service life, since no significant degradation was found even after aging in actual service conditions for the periods as long as 4.8 years and at depths deeper than 2000 m.

## 5. Conclusions

This study found a small effect of aging, in actual operation conditions of ESP cables, on the mechanical, thermal, structural and barrier properties of the two EPDM-based layers (insulation and jacket).

It is suggested that the thermo-oxidation mechanism followed by chain scission does not have a role in the degradation of EPDM within the aged ESP cables, and no sign of increase or decrease in crosslink fraction has been encountered.

Hardness of EPDM hardly changed in both layers during natural aging in actual operation conditions of ESP cables.

While EPDM from the insulation layer did not present changes in barrier properties on aging, EPDM from the jacket layer of the cables A and B (2 years of aging at 917 m and 1927 m, respectively) showed slightly lower barrier properties than the EPDM from the cables C and D (4.8 years of aging at 760 and 2170 m, respectively) and from the virgin cable E. This difference was attributed to possibly different chemical environments in two different wellbores, since the cables A and B served in a different wellbore from the cables C and D.

Activation energies of degradation calculated by the Kissinger method indicated a modest decrease (up to 10%) only for the EPDM from jacket layers.

The previous data pointed out that slight degradation of EPDM from the jacket layer, detected mainly through a reduction of activation energy, is due to some non-oxidation mechanism, such as, for example, chemical degradation and/or hydrolysis. To complement the data obtained in this work, it is intended to perform nuclear magnetic resonance spectroscopy of virgin and aged EPDM samples.

It can be concluded from our study that EPDM-based layers from ESP cables seem not to be weak links in the structure of modern ESP systems, although a higher number of ESP cables from different wellbores, naturally aged in actual operation conditions during periods of several years, and at different depths, would be a mandatory requirement to acquire a more robust data set and knowledge on degradation processes inside these cables. In addition, there are no current acceptance criteria for the lifespan of ESP cable parts and, therefore, these need to be established.

## Figures and Tables

**Table 1:** Vibrational bands of EPDM.

Band (cm^−1^)	Interpretation	Description
3075	$\upsilon_{\mathrm{sy}}{\mathrm{SP}}^{2}=C-H$	Symmetrical stretch C–H
2957	$\upsilon_{\mathrm{asy}}{\mathrm{SP}}^{3}\;C-H$	Asymmetric stretch C–H
2915	$\upsilon_{\mathrm{asy}}{\mathrm{SP}}^{3}\;C-H_{3}$ $\upsilon_{\mathrm{asy}}{\mathrm{SP}}^{3}\;C-H_{2}$	Asymmetric stretch C–H
2848	$\upsilon_{\mathrm{sy}}{\mathrm{SP}}^{3}\;C-H_{3}$ $\upsilon_{\mathrm{sy}}{\mathrm{SP}}^{3}\;C-H_{2}$	Symmetrical stretch C–H
1455	$\delta_{\mathrm{asy}}{\mathrm{SP}}^{3}\;C-H_{3}$ $\delta_{\mathrm{sy}}{\mathrm{SP}}^{3}\;C-H_{2}$	Asymmetric angular deformation in the plane CH_3_Symmetrical angular deformation in plane (scissor) CH_2_
1374	$\delta_{\mathrm{sy}}{\mathrm{SP}}^{3}\;C-H_{3}$	Symmetrical angular deformation in the plane (umbrella) CH_3_
709	$\delta_{\rho }{\mathrm{SP}}^{3}{\;\mathrm{CH}}_{2}$	Asymmetric angular deformation off plane (wagging) $n_{{\mathrm{CH}}_{2}}\geq 4$

**Table 2:** Weight losses of the insulation layer for the cables A–E evaluated under condition I.

Cable	%Weight Loss 30–250 °C	%Weight Loss 250–410 °C	%Weight Loss 410–570 °C
A	0.3	2.9	59.7
B	0.3	2.9	59.8
C	0.3	3	59.7
D	0.9	3.3	58.7
E	0.3	2.8	59.4
**Average**	**0.4**	**3**	**59.5**
**Standard deviation**	**0.2**	**0.2**	**0.4**

**Table 3:** Activation energies of the insulation and jacket layers, as calculated by the Kissinger method.

E_a_ (kJ mol^−1^)		
Cable	Insulation Layer	Jacket Layer
A	227.20	227.20
B	242.00	217.89
C	235.48	217.96
D	235.49	216.94
E	232.93	237.83

**Table 4:** Weight losses of the jacket layer for the cables A–E evaluated under condition I.

Cable	%Weight Loss 30–250 °C	%Weight Loss 250–410 °C	%Weight Loss 410–570 °C
A	0.7	13.6	54.6
B	0.6	13.8	54.1
C	1.4	13.1	55.8
D	1.6	14.7	56.3
E	1.4	14.1	55.8
**Average**	**1.2**	**13.9**	**55.3**
**Standard deviation**	**0.4**	**0.5**	**0.8**

**Table 5:** Weight losses and residues for the cables A–E, evaluated under condition II.

**Cable**	**% of Weight Loss from 570 to 930 °C (Carbon Black)**				
	A	B	C	D	E
	17.06	17.35	18.48	18.57	18.46
**Cable**	**% of Weight Residue at 930 °C (A, B: Kaolinite and C, D, E: Metakaolinite)**				
	A	B	C	D	E
	13.86	14.07	11.61	8.59	9.89

**Table 6:** Crosslink fractions for insulation and jacket layers.

Cable	Insulation Layer	Standard Deviation	Jacket Layer	Standard Deviation
A	0.91	0.005	0.65	0.03
B	0.92	0.02	0.72	0.02
C	0.91	0.03	0.72	0.04
D	0.84	0.02	0.73	0.006
E	0.87	0.02	0.69	0.02

## Data Availability

The data underlying this article will be shared on reasonable request from the corresponding author.

## Supplementary Materials

The following are available online at https://www.mdpi.com/article/10.3390/ma14195520/s1, Figure S1: Schematic draw of ESP cable configuration, with its principle layers.
