# Supplementary material for: Natural Aging of Ethylene-Propylene-Diene Rubber under Actual Operation Conditions of Electrical Submersible Pump Cables
Source: Materials (Basel). 2021 Sep 24;14(19):5520. doi: 10.3390/ma14195520 (PMC8509601; doi:10.3390/ma14195520)
Supplement: Supplementary file 1 [file materials-14-05520-s001.zip › materials-1324305-supplementary.pdf]

materials

MDPI

Supplementary Materials: Natural Aging of Ethylene-Propylene-Diene Rubber under Actual Operation Conditions of Electrical Submersible Pump Cables

Freddy Ignacio Rojas Rodríguez, José Roberto Moraes d'Almeida and Bojan A. Marinkovic *

Department of Chemical and Materials Engineering, Pontifical Catholic University of Rio de Janeiro (PUC-Rio), Rio de Janeiro 22453-900, Brazil; freddyrojas@aluno.puc-rio.br (F.I.R.R.); dalmeida@puc-rio.br (J.R.M.d.)

* Correspondence: bojan@puc-rio.br; Tel.: +55-21-3527-1954

Citation: Rojas Rodriguez, F.I.; d'Almeida, J.R.M.; Marinkovic, B.A. Natural Aging of Ethylene-Propylene-Diene Rubber under Actual Operation Conditions of Electrical Submersible Pump Cables. *Materials* **2021**, *14*, 5520. https://doi.org/10.3390/ma14195520

Academic Editor: Yi Gong

Received: 18 July 2021
Accepted: 17 September 2021
Published: 24 September 2021

Publisher's Note: MDPI stays neutral with regard to jurisdictional claims in published maps and institutional affiliations.

Copyright: © 2021 by the authors. Submitted for possible open access publication under the terms and conditions of the Creative Commons Attribution (CC BY) license (http://creativecommons.org/licenses/by/4.0/).

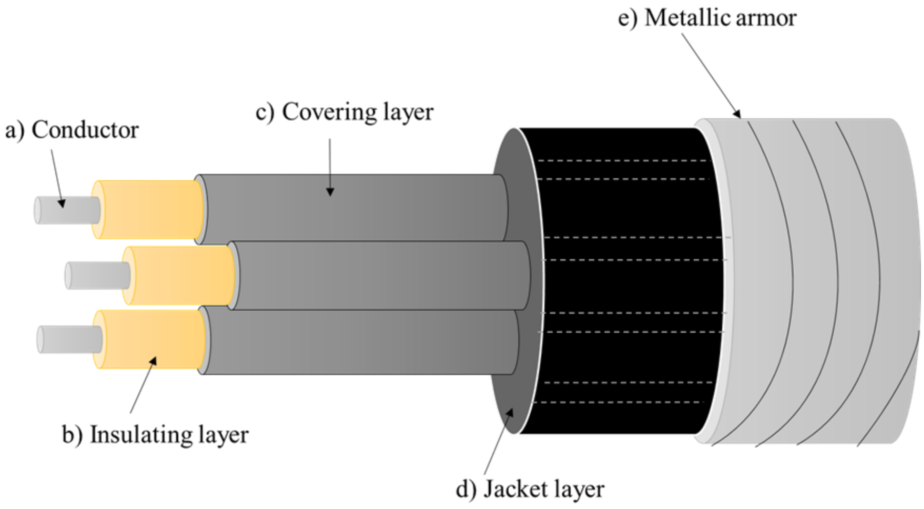

Figure S1. Schematic draw of ESP cable configuration, with its principal layers.
